# Innate Immune Responses Are Increased in Chronic Obstructive Pulmonary Disease

**DOI:** 10.1371/journal.pone.0018426

**Published:** 2011-03-31

**Authors:** Katherine Joanne Baines, Jodie Louise Simpson, Peter Gerard Gibson

**Affiliations:** 1 Priority Research Centre for Asthma and Respiratory Diseases, The University of Newcastle, Callaghan, New South Wales, Australia; 2 Department of Respiratory and Sleep Medicine, Hunter Medical Research Institute, John Hunter Hospital, New Lambton, New South Wales, Australia; Ludwig-Maximilians-Universität München, Germany

## Abstract

**Background:**

Chronic obstructive pulmonary disease (COPD) is characterised by irreversible airflow obstruction, neutrophilic airway inflammation and chronic bacterial colonisation, however the role of the innate immune response in the pathogenesis of COPD remains unclear.

**Methods:**

Induced sputum was obtained from adults with COPD (n = 22), and healthy controls (n = 29) and was processed for differential cell counts. The sputum supernatant was assayed for innate immune mediators using ELISA, whilst sputum gene expression was measured using real-time PCR. Peripheral blood neutrophils were isolated and their response to lipopolysaccaride (LPS) stimulation was assessed in a subgroup of participants with COPD (n = 13) and healthy controls (n = 21).

**Results:**

Participants with COPD had significantly higher protein levels of interleukin (IL)-8, and neutrophil elastase (NE) and detection of oncostatin M (OSM) compared to healthy controls. Gene expression for toll-like receptor (TLR) 2, IL-8 and OSM were also significantly higher in COPD participants. The level of IL-1β, surfactant protein (SP)-A, matrix metalloproteinase (MMP)-9 and TLR4 mRNA was not significantly different between groups. The level of innate immune response markers were highly associated with the presence of sputum neutrophils, each other and the degree of airflow limitation (FEV_1_/FVC). Peripheral blood neutrophils from participants with COPD had an increased response to stimulation by LPS; with a greater fold increase in the production of IL-8 and MMP-9 protein, and gene expression of IL-8, TLR2 and TLR4.

**Conclusions:**

The innate immune response is increased in the airways and circulating neutrophils in COPD, and may be an important mechanism involved in disease pathogenesis.

## Introduction

Chronic obstructive pulmonary disease (COPD) is responsible for a major and increasing burden of illness and death around the world. It is currently the fourth leading cause of death in most industrialised countries, and by the year 2020 it is predicted to be the third leading cause of death worldwide [1]. COPD is chronic and progressive, is characterised by incompletely reversible airflow obstruction, symptoms of dyspnoea, cough and sputum production, and an abnormal inflammatory response involving neutrophils, macrophages and CD8^+^ T lymphocytes in response to noxious particles such as cigarette smoke [2], [3].

The innate immune response in the airways involves the detection of pathogen- or damage-associated molecular patterns (PAMPs or DAMPs), by pattern recognition receptors (PRRs) such as toll-like receptors (TLRs) on the cell surface and secreted receptors such as the collectins such as surfactant proteins [4]. Activation of PRRs triggers a signalling cascade leading to the activation of nuclear factor kappa-light-chain-enhancer of activated B cells (NF-κB), resulting in the production of inflammatory chemokines and cytokines [5], [6]. Triggers of the innate immune response, including infection by bacteria or viruses, and environmental exposures such as cigarette smoke and air pollution, are common exposures in people with COPD. Persistent innate immune activation has been linked to chronic inflammatory airway diseases such as neutrophilic asthma, bronchiectasis [7] and models of chronic airway disease [8]. This activation is thought to be caused by the interaction of PRRs with viruses, bacteria, reactive oxygen species and dead and damaged cells and leads to the development and exacerbations of COPD [9], [10].

The presence of neutrophils in the airways is increased in COPD [3], [11] and associated with increased levels of neutrophilic inflammatory mediators including interleukin (IL)-8 [12]. Markers of airway neutrophilic inflammation are correlated with COPD disease progression [13], clinical severity [14] and exacerbations [15]. Peripheral blood neutrophils show altered activity in both stable COPD and during exacerbations, including increased expression of cell surface adhesion molecules [16]–[18], upregulation of genes relating to inflammation [19] and enhanced respiratory burst [17].

While airway inflammation in COPD has been well characterised, the inflammatory mechanisms resulting in this chronic and destructive neutrophilic inflammation are not well understood. Since COPD has been proposed as an ‘archetypal disease of innate immunity’ [9] this study investigated the innate immune response in both the airways and peripheral blood neutrophils of participants with COPD compared to their healthy counterparts. We hypothesised that people with COPD would express higher levels of innate immune mediators in the airway, and show features of systemic involvement with an increased response of circulating neutrophils to innate immune stimulation with the TLR4/2 agonist, lipopolysaccharide (LPS). To examine these effects, we have examined a broad range of innate markers, in both the systemic and airway compartments, examined TLR signalling in response to the TLR4 agonist LPS, and related these changes to smoking status and airflow obstruction.

## Methods

### Ethics Statement

The Hunter Area Health Service and The University of Newcastle Research Ethics Committee's approved this study. All participants gave informed written consent prior to their inclusion in this study.

### Subjects and Design

Participants with COPD(n = 22) had a clinical diagnosis of symptomatic COPD. Healthy controls without respiratory disease (n = 29) were over the age of 40 years, had an FEV_1_>80% of predicted and were matched for smoking status. Exclusion criteria included a current or recent (past month) respiratory tract infection, exacerbation of respiratory disease, or a course of oral steroids or antibiotics in the previous month. Participants were recruited from the Respiratory Ambulatory Care Service at John Hunter Hospital and by advertisement.

### Clinical Assessments

Participants underwent clinical assessment, spirometry, combined hypertonic saline challenge and sputum induction, allergy skin prick testing, and in some cases blood collection. Information was collected regarding smoking history, and the St George Respiratory Questionaire was completed to assess quality of life. The carbon monoxide transfer co-efficient (KCO) was determined according to ATS guidelines (Med-Graphics DX Pulmonary function testing system, Medical Graphics Corporation, Minnesota, MN, USA) [20].

### Sputum Induction and Analysis

Spirometry (KoKo PD Instrumentation, Louisville, CO, USA) and combined bronchial provocation testing and sputum induction with hypertonic saline (4.5%) were performed as previously described [21]. 100 µL of selected sputum was transferred to RLT buffer (Qiagen, Hilden, Germany) and stored at −80°C. An aliquot of sputum was used for bacteriological culture, and bacterial identification was determined by the Hunter Area Pathology Service. The remaining selected sputum was dispersed using dithiothreitol as described [21]. The suspension was filtered, and a total cell count (TCC) of leucocytes and viability performed. Sputum supernatant was was stored at −80°C, until mediator analysis and cytospins were prepared from the cell pellet, stained and a differential cell count obtained from 400 non-squamous cells.

### Detection of Mediators

The concentrations of IL-8, IL-1β, TNF-α, OSM and Total MMP-9 protein were determined by ELISA (R&D Systems, Minneapolis, MN, USA), and NE by the Innozyme Human Neutrophil Elastase Immunocapture Activity Assay Kit (Calbiochem, La Jolla, CA, USA) as per manufacturer's instructions. Target gene expression was analysed using quantitative real-time PCR as described previously [22]. Briefly, RNA was extracted and reverse-transcribed to cDNA. Taqman qPCR probes for IL-8, OSM, TLR2 and TLR4 mRNA were purchased in kit form, combined with the reference gene eukaryotic 18S ribosomal RNA in duplex real-time PCRs (ABI 7500 Real Time PCR Machine, Applied Biosystems, Foster City, CA, USA) and results calculated using 2^-ΔΔCt^ relative to the housekeeping gene (18S) and an internal calibrator.

### Peripheral Blood Neutrophil Culture

Peripheral blood neutrophils were isolated using Percoll density gradient and magnetic cell separation as previously described [23]. Cells were resuspended in RPMI1640 (Gibco Invitrogen, Mount Waverley, Australia) with 10 mM HEPES, 1% fetal calf serum and antibiotics (Penicillin/Streptomycin). Cells were cultured at 1×10^6^cells/ml +/− LPS (10 or 100 ng/ml E.coli LPS, Sigma, Sydney, Australia) at 37°C (5% CO_2_) for 24 hours before cell free supernatants were prepared and cell pellets were and stored in RLT buffer (Qiagen, Hilden, Germany) at −80°C until further analysis.

### Data Analysis

Data were analysed using Stata 9 (Stata Corporation, College Station, Texas USA), with results reported as median and interquartile range unless otherwise indicated. Analysis was performed using the two-sample Wilcoxon Rank Sum test. Chi squared or fischers' exact tests was applied to analyse categorical data. Associations between data were determined using Spearman's rank correlation. Results were reported as significant when p<0.05.

## Results

### Clinical parameters

Participants with COPD were older and had considerably lower lung function, but were similar to healthy controls in terms of gender and atopy (Table 1). About half of participants with COPD (n = 10, 45%) were taking inhaled corticosteroid therapy (2000 (2000, 2000) µg beclomethasone equivalents/day). COPD severity was classified as mild (stage I, n = 3 (14%)), moderate (stage II, n = 17 (77%)), and severe (stage III, n = 2 (9%)) according to GOLD criteria. Smoking history and pack years smoked were significantly greater in the COPD group (Table 1).

**Table 1:** Clinical characteristics of subjects with COPD and healthy control subjects.

	Healthy Controls	COPD	p
**N**	29	22	
**Age years, mean (SD)**	59 (10)	67 (7)	0.001
**Gender M | F**	12 | 17	15 | 7	0.058
**Body Mass Index, mean (SD)**	27.0 (6.1)	28.4 (5.7)	0.414
**Atopy n (%)**	17 (59)	11 (50)	0.540
**Smoking, Never | Ex | Current**	12 | 12 | 5	2 | 17 | 3	0.021
**Pack years, median (Q1, Q3)**	13 (4, 35)	63 (31, 75)	0.001
**FEV_1_% predicted, post bronchodilator, mean (SD)**	99 (11)	67 (11)	<0.001
**FVC% predicted, post bronchodilator, mean (SD)**	78 (12)	53 (9)	<0.001
**FEV_1_/FVC %, mean (SD)**	76 (5)	61 (8)	<0.001
**FEF 25–75% predicted, post bronchodilator, mean (SD)**	76 (24)	33 (9)	<0.001
**KCO % Predicted, mean (SD)**	79.5 (12.6)	63.3 (20.3)	<0.001
**Quality of Life Score, total, median (Q1,Q3)**	4 (1, 5)	35 (16, 46)	<0.001
**Culture positive n (%)**	0 (0)	3 (14)	0.037

### Inflammatory Cells

Participants with COPD had a significantly increased total cell count, proportion and number of neutrophils and eosinophils (Table 2). The proportion of macrophages was lower in participants with COPD; however numbers of macrophages did not differ between groups.

**Table 2:** Inflammatory cell counts for participants with COPD and healthy controls.

	Healthy Controls	COPD	p
**n**	25	19	
**Total cell count × 10^6^/mL**	2.1 (1.6, 4.3)	4.9 (2.8, 10.7)	<0.001
**Viability**	84.3 (72.7, 88.5)	88.7 (80.7, 91.3)	0.267
**Neutrophils, %**	36.0 (24.5, 47.8)	53.8 (35.8, 68.0)	0.011
**Neutrophils 10^4^/mL**	92.9 (32.1, 173.5)	197.9 (147.9, 590.0)	0.005
**Eosinophils, %**	0.1 (0.0, 0.5)	0.8 (0.3, 2.3)	0.013
**Eosinophils 10^4^/mL**	0.2 (0.0, 1.2)	4.5 (1.3, 12.6)	<0.001
**Macrophages, %**	57.8 (48.0, 66.8)	41.3 (27.8, 48.5)	0.003
**Macrophages 10^4^/mL**	125.7 (85.4, 216.0)	171.7 (115.1, 288.0)	0.108
**Lymphocytes, %**	1.5 (0.4, 3.1)	1.0 (0.3, 2.0)	0.398
**Lymphocytes 10^4^/mL**	3.7 (1.1, 6.2)	4.2 (1.2, 12.0)	0.405
**Columnar epithelial cells, %**	1.3 (0.6, 3.1)	1.3 (0.8, 2.8)	0.704
**Columnar epithelial cells 10^4^/mL**	2.6 (1.4, 7.2)	6.0 (1.8, 16.7)	0.382
**Squamous cells, %**	3.0 (1.1, 6.7)	1.7 (0.2, 2.9)	0.086

Results shown are median (Q1, Q3).

### Airway Innate Immune Mediators

**Figure 1:**
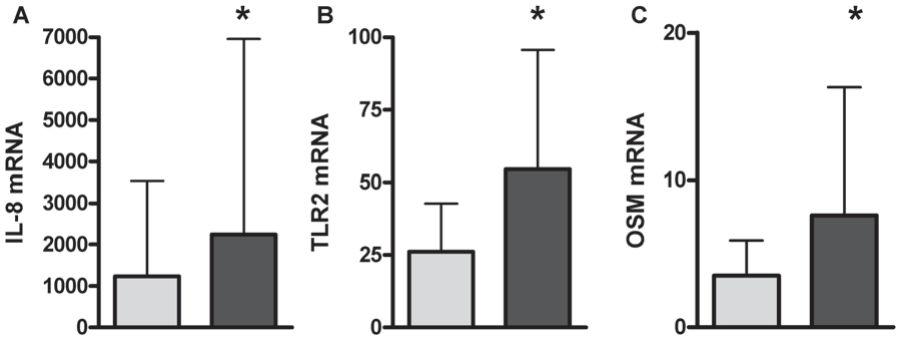
Gene expression of IL-8 (A), TLR2 (B) and OSM (C) was increased in the airways of participants with COPD (dark grey) compared to healthy controls (light grey). Data reported as median with the error bar as the 3^rd^ quartile. *p<0.05 versus healthy controls.

**Figure 2:**
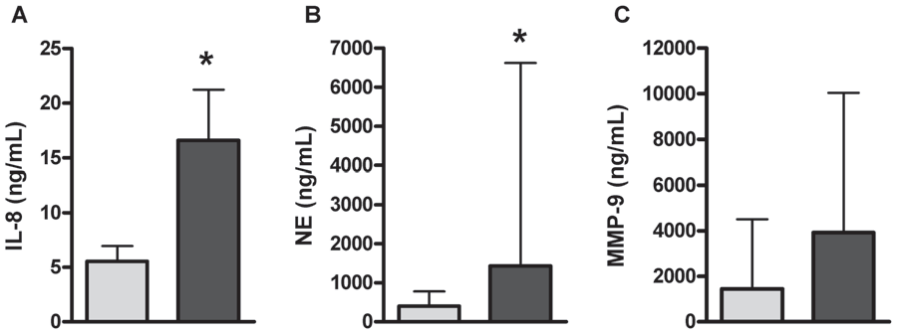
IL-8 (A), HNE (B) and MMP-9 (C) protein levels were higher in the airways of participants with COPD (dark grey) compared to healthy controls (light grey). Data reported as median with the error bar as the 3^rd^ quartile. *p<0.05 versus healthy controls.

OSM protein was not detectable in healthy participants, however OSM was detected significantly more in COPD with 26% (n = 5; p = 0.046; 1349.9(867.8, 1820.1) pg/mL) of samples at detectable levels. Gene expression of TLR2 (Figure 1A; p = 0.021), IL-8 (Figure 1B; p = 0.041) and OSM (Figure 1C; p = 0.034) were significantly increased in COPD, whereas gene expression for TLR4 [Healthy: 5.8(4.3–7.5); COPD: 6.7(4.9–8.4); p = 0.621] remained unchanged. Participants with COPD had significantly higher levels IL-8 (Figure 2A; p≤0.001) and NE (Figure 2B; p = 0.015) protein, and higher levels of total MMP-9, however this did not reach statistical significance (Figure 2C; p = 0.168). The level of SP-A [Healthy: 47.9 (34.8–96.8); COPD: 50.5 (44.6–98.7); p = 0.285] or IL-1β [Healthy: 15.7 (1.6–39.3); COPD: 35.7 (15.3–71.3); p = 0.236] protein did not differ between participants with COPD and healthy controls. A sub analysis of participants taking inhaled corticosteroid (ICS) compared to those not taking ICS showed that ICS use did not alter airway mediator levels in COPD.

### Associations

**Figure 3:**
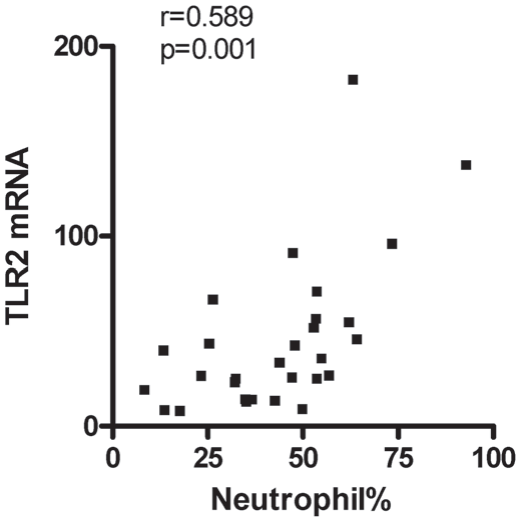
Sputum neutrophil % and TLR2 gene expression are significantly correlated (Spearman R = 0.59; p = 0.001).

Strong correlations were observed between markers of the innate immune response including sputum neutrophils, TLR2 gene expression, IL-8, MMP-9 and NE protein (Table 3). There was a positive correlation between sputum neutrophil % and the gene expression of TLR2 (r = 0.59; Figure 3). There were significant negative correlations between measures of airflow obstruction and inflammatory markers. FEV_1_/FVC was significantly negatively correlated with sputum neutrophil% (r = −0.34; p = 0.033), sputum neutrophil number (r = −0.33; p = 0.037), level of IL-8 (r = −0.49; p = 0.002) and NE (r = −0.41; p = 0.012). In addition, FEV_1_% predicted was significantly negatively correlated with the level of IL-8 (r = -0.42; p = 0.009).

**Table 3:** Correlation matrix for innate immune response markers.

	Neutrophil, %	TLR2 mRNA	Total MMP-9	IL-8 ng/mL	NE ng/mL
Neutrophils 10^4^/mL	0.82^c^	0.38	0.56^b^	0.74^c^	0.71^c^
Neutrophil, %		0.59^b^	0.65^c^	0.61^c^	0.66^c^
TLR2 mRNA			0.38	0.32	0.43^a^
Total MMP-9				0.55^b^	0.62^b^
IL-8 ng/mL					0.79^c^

Spearman

a p<0.05,

b p<0.001,

c p<0.0001.

### Impact of Smoking Status

The impact of smoking status was investigated by repeating the analysis of innate immune response markers after removal of all participants that were never smokers. This did not change the outcomes measured. Ex and current smoking participants with COPD had significantly more OSM detected (p = 0.036), significantly higher gene expression of TLR2 (p = 0.019), IL-8 (p = 0.030) and OSM (p = 0.028), and significantly higher levels of IL-8 (p<0.001), NE (p = 0.013) and higher levels of MMP-9 (p = 0.13) compared to healthy ex and current smokers. However, there was positive correlation between the degree of smoking history (pack years smoked) with the number of airway neutrophils (r = 0.39; p = 0.024); the level of IL-8 ng/ml (r = 0.51; p = 0.003) and NE ng/mL (r = 0.49; p = 0.005); and negative correlation with FEV_1_% predicted (r = −0.37; p = 0.026) and FEV_1_/FVC (r = −0.45; p = 0.005).

### Peripheral Blood Neutrophil Responses

**Figure 4:**
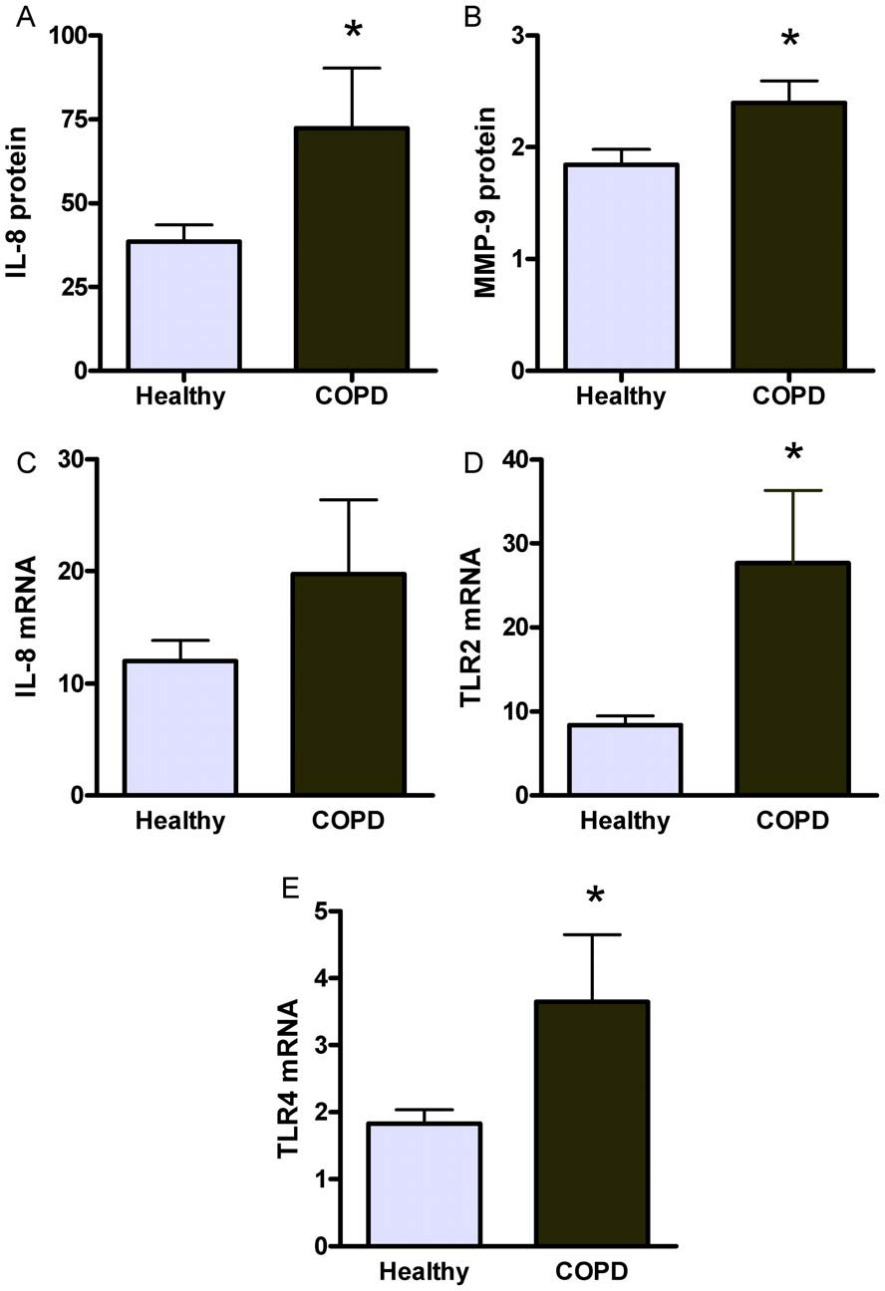
Peripheral blood neutrophils isolated from participants with COPD (dark grey) had a greater increase in the production of IL-8 (A) and MMP-9 (B) protein and the gene expression of IL-8 (C), TLR2 (D), and TLR4 (E) in response to LPS stimulation compared to healthy controls (light grey). Data expressed as fold increase versus media control.*p<0.05 versus healthy controls.

Peripheral blood neutrophils isolated from participants with COPD had exaggerated innate immune responses to stimulation with LPS. There were higher fold increases observed for IL-8 and MMP-9 protein, and IL-8, TLR2 and TLR4 mRNA (Figure 4; Clinical details of participants are shown in Table 4).

**Table 4:** Clinical details of participants with COPD and healthy controls in whom in vitro LPS responses of peripheral blood neutrophils were assessed.

	Healthy	COPD	p
**N**	21	13	
**Age years, mean (SD)**	56 (20)	67 (6)	0.065
**Gender M | F**	10 | 11	6 | 7	0.934
**FEV1% predicted, mean (SD)**	103 (13)	61 (16)	<0.001
**FEV1/FVC %, mean (SD)**	86 (11)	68 (16)	<0.001

## Discussion

This study investigated the innate immune response of neutrophils in both the airway and systemic compartments in participants with COPD and examined responses in relation to the degree of airflow obstruction. In airway samples from COPD participants, there was increased constitutive expression of innate immune response markers, and in the systemic compartment there was an increased innate immune response of circulating neutrophils to a TLR4 agonist. Gene expression for TLR2 was increased in airway samples in COPD, and there was enhanced TLR2 and TLR4 gene expression in circulating neutrophils with LPS stimulation, demonstrating the potential for chronic activation of innate immune responses. The consequences of this was increased levels of IL-8, NE, MMP-9 and OSM protein detected in the airways of participants with COPD as well as an upregulation of IL-8, OSM, TLR2 gene expression. The innate immune mediators IL-8, NE, MMP-9 and TLR2 were highly correlated with airway neutrophils and each other, suggesting a positive feedback cycle of neutrophilic airway inflammation [24]. This cycle is likely to impact on lung function and particularly the degree of airflow limitation, as significant correlations were observed between FEV_1_/FVC with sputum neutrophils, IL8 and NE protein. Furthermore, in COPD, systemic neutrophils showed a significantly increased response to in vitro LPS stimulation in the way of IL-8, and MMP-9 production.

These results indicate that the innate immune response is active both systemically and in the airways in stable COPD. The presence of increased numbers of neutrophils in the airways of COPD patients together with increased levels of neutrophilic inflammatory mediators in airway samples, including cytokines such as IL-8 and TNF-α, and proteases such as NE and MMP-9 [12], [25], [26] has been observed previously. Our study confirms the increased production of IL-8 and NE, and makes the novel observation that OSM, an IL-6 family cytokine, was detected more frequently in the airways of COPD patients. OSM plays an important role in airway remodelling in asthma [27], and has been shown to be increased in asthma and airway obstruction [28]. Interestingly, IL-1β was not elevated, and has recently been shown to be suppressed by cigarette smoke exposure [29].

TLRs present on neutrophils are thought to mainly be involved in antibacterial responses, including the recognition of gram positive bacteria by TLR2 [30], and gram negative bacteria by TLR4 [4] and bacterial DNA by TLR-9 [31]. Activation of TLR2, TLR4 and TLR9 regulates several important neutrophil functions through the activation of the NF-κB pathway, including neutrophil activation, migration and survival [32]. We have previously reported an upregulation of TLR2 associated with neutrophilic airway inflammation in neutrophilic asthma [7]. We now show that TLR2 mRNA is upregulated in COPD, and that TLR2 and TLR4 expression increase dramatically upon LPS stimulation of blood neutrophils. This indicates that these cells upon stimulation would have an increased capacity to respond to innate immune triggers important in COPD pathogenesis.

Cigarette smoking is widely recognised as a primary risk factor for the development of COPD, and participants with COPD in our study had a greater history of smoking than our control group. Components of cigarette smoke can cause an inflammatory response upon inhalation and this exposure is considered to be the starting point disease pathogenesis in COPD [33]. We found that smoking dose was positively correlated with inflammatory mediator levels. It is not fully understood how the innate immune system responds to cigarette smoke, however it has been proposed that the injury to the airway epithelium produces ‘danger signals’ or DAMPs that can act as ligands for TLRs including TLR2, TLR4 [34] and TLR9 [31]. This results in the activation of NF-κβ and the production of inflammatory mediators that attract neutrophils and macrophages [35]. The inflammatory response caused by exposure of mice to cigarette smoke has been reported to be dependent on both TLR4 and IL1R1 signalling involving the associated protein myeloid differentiation factor 88 (MyD88) [36].

The inflammatory response in COPD is not limited to the lungs, but may also be seen in the systemic compartment [37]. We have demonstrated that blood neutrophils in COPD have an increased response to in vitro LPS stimulation, with increased production of IL-8, OSM, and release of MMP-9. This enhanced response could contribute significantly to the systemic inflammation seen in COPD, as well as the migration of cells to the pulmonary compartment. Other studies have shown an enhanced activation of systemic neutrophils in COPD, particularly a potentiation of migratory and cytotoxic responses [16]–[18], but also upregulation of inflammatory genes [19]. We have previously shown that alterations in cytokine production and gene expression of circulating neutrophils are important in non-eosinophilic asthma [38], which is also associated with neutrophilic airway inflammation.

Interestingly, airway levels of IL-8, NE, MMP-9, TLR2 mRNA and neutrophils were highly correlated, indicating that these mediators are both associated with neutrophilic airway inflammation and each other. We have previously proposed that the production of IL-8, NE and MMP-9 occurs in a positive feedback cycle that leads to persistence of neutrophilic inflammation in the airways, and that this is postulated to be due to amplification of the innate immune response involving stimulation of TLR2 [24]. The data reported here further corroborate these findings, and show similarities between neutrophilic inflammation seen in COPD as to that previously reported in neutrophilic asthma, suggesting a common mechanism [7], [39]. The data also suggest a common pathogenic network may be operating to mediate the tissue responses seen in COPD [40]. Insight into the role of the innate immune response is of high relevance for the identification of further diagnostic and therapeutic approaches. Development of treatments targeted at reducing neutrophilic airway inflammation would be of great benefit for the treatment of COPD [9].

Neutrophils are key effector cells in COPD, and our observations that the presence of airway neutrophils and the level of IL-8 and NE relate to the severity of airflow obstruction extend these observations. We have also shown that the number of pack years smoked is negatively correlated with the severity of airflow obstruction and positively correlated with the accumulation of airway neutrophils. This suggests that cigarette smoking can influence the accumulation of airway neutrophils, which is associated with production of innate immune mediators and an increase in airflow obstruction.

In summary, we have shown that participants with COPD had significantly higher levels of airway IL-8, NE, OSM, and TLR2 mRNA and an increased release of IL-8, MMP-9 and OSM protein, and gene expression of IL-8, OSM, TLR4 and TLR2 from peripheral blood neutrophils in response to in vitro TLR stimulation. IL-1β and SP-A were not implicated in this response. This increased innate immune response both in the airways and peripheral blood neutrophils in COPD further implicates the activation of the innate immune response as an important mechanism of disease pathogenesis. Both bacterial colonisation and cigarette smoking are likely triggers of this aberrant neutrophilic response.
